# Comparative Analysis of IPSS, IPSS-R, and WPSS for Predicting Survival and Leukemic Transformation in Myelodysplastic Neoplasms: A Real-World Single-Center Experience

**DOI:** 10.3390/jcm14165757

**Published:** 2025-08-14

**Authors:** Mihai-Emilian Lapadat, Oana Stanca, Nicoleta Mariana Berbec, Silvana Angelescu, Irina Nicoleta Triantafyllidis, Anca Mariana Ciobanu, Cristina Negotei, Cristian Tudor Barta, Georgian Halcu, Carmen Saguna, Constanta Elena Popovici, Ana-Maria Bordea, Madalina Marilena Oprea, Andrei Colita

**Affiliations:** 1 Department of Hematology, “Carol Davila” University of Medicine and Pharmacy, 050474 Bucharest, Romania; mihai-emilian.lapadat@umfcd.ro (M.-E.L.); oana.stanca@umfcd.ro (O.S.); nicoleta.berbec@umfcd.ro (N.M.B.); silvana.angelescu@umfcd.ro (S.A.); irina.triant@yahoo.com (I.N.T.); cristina.negotei@umfcd.ro (C.N.); cristian.barta@umfcd.ro (C.T.B.); georgian.halcu@drd.umfcd.ro (G.H.); carmen.saguna@umfcd.ro (C.S.); elena.coles@umfcd.ro (C.E.P.); 2 Clinic of Hematology, Coltea Clinical Hospital, 030171 Bucharest, Romania; ancaciobanu05@yahoo.com (A.M.C.); ana_maria_ivanescu@yahoo.co.uk (A.-M.B.); mady_1969@yahoo.com (M.M.O.)

**Keywords:** myelodysplastic syndrome, IPSS, IPSS-R, WPSS, risk stratification, survival, leukemic transformation

## Abstract

**Background:** Myelodysplastic syndromes are clonal hematopoietic disorders characterized by ineffective hematopoiesis and risk of progression to acute myeloid leukemia. Accurate prognostic stratification is essential to guide treatment, with several scoring systems in clinical use: IPSS, IPSS-R, and WPSS. **Objective:** We aimed to evaluate the prognostic accuracy of IPSS, IPSS-R, and WPSS in a real-world Romanian MDS cohort by comparing risk classifications with observed overall survival and progression-free survival. **Methods:** We conducted a retrospective analysis of 117 patients diagnosed with MDS treated in our clinic between 2018 and 2022. All patients had confirmed diagnoses based on bone marrow biopsy and cytogenetic testing. Data were used to assign risk categories based on IPSS, IPSS-R, and WPSS. Survival outcomes were analyzed using Kaplan–Meier curves and log-rank tests. **Results:** The median age of the cohort was 70 years; gender distribution was balanced. Transfusion dependence was present in 73.5%, and 49.6% had cytogenetic abnormalities. Overall, low-risk classification was assigned in 58.1% (IPSS), 38.5% (IPSS-R), and 38.5% (WPSS) of patients. Median OS was 20 months, and median PFS was 35 months. Although no statistically significant overall survival differences were observed across scoring systems, IPSS-R demonstrated a trend toward stronger prognostic discrimination in multivariable analysis. Reclassification of patients initially categorized as IPSS intermediate-1 revealed a significant survival impact: patients reclassified as lower-risk by IPSS-R and WPSS had a median OS of 67.5 months versus 15 months for those reclassified as higher-risk (IPSS-R: HR = 0.24; *p* = 0.0017; WPSS: HR = 0.26; *p* = 0.0031). Similarly, leukemic transformation occurred in 13.6% of reclassified lower-risk patients vs. 52.2% in higher-risk patients (IPSS-R: HR = 0.13; *p* = 0.0021; WPSS: HR = 0.12; *p* = 0.002), with a median PFS of 21 months in the higher-risk group. In multivariable Cox regression analysis, IPSS-R stratification remained a strong independent predictor for both OS (HR = 3.22; *p* = 0.000003) and PFS (HR = 4.77; *p* < 0.00001), while azacitidine treatment was associated with significantly improved survival (OS: HR = 0.43; *p* = 0.00002) and reduced risk of progression (PFS: HR = 0.36; *p* = 0.013).

## 1. Introduction

Myelodysplastic neoplasms or myelodysplastic syndromes (MDS) are a group of clonal myeloid malignancies characterized by dysplastic hematopoiesis, cytopenias, and a risk of progression to acute myeloid leukemia (AML) [1,2,3]. These disorders arise from genetic and epigenetic alterations within the hematopoietic stem cell (HSC) pool, leading to clonal hematopoiesis [1] and expansion of progenitor populations with morphological abnormalities (dysplasia) that exhibit increased rates of apoptosis. The result is peripheral blood cytopenias such as neutropenia, anemia, or thrombocytopenia [2]. With disease evolution, additional mutations may accumulate, leading to increased blast proliferation and the likelihood of transformation to AML [3]. Secondary MDS can also arise following exposure to prior radiotherapy or chemotherapy for other malignancies [4].

One of the defining features of MDS is its marked biological and clinical heterogeneity. This has driven ongoing efforts to develop reliable prognostic models for risk stratification, guiding therapeutic decision-making and predicting clinical outcomes [5,6,7,8,9]. Several prognostic scoring systems have been developed for this purpose, among which the International Prognostic Scoring System (IPSS) [5], its revised version (IPSS-R) [6], and the WHO Prognostic Scoring System (WPSS) [7] are most widely used. These systems utilize different prognostic factors to stratify patients in distinct risk categories. A more recent model, the Molecular International Prognostic Scoring System (IPSS-M) [8], incorporates mutational status of 31 genes associated with MDS, allowing for more refined patient stratification. Available data demonstrate the superior accuracy of IPSS-M [10,11,12,13], particularly among older patients [14]. However, due to limited access to molecular diagnostics in many settings, this tool is not yet widely adopted in clinical practice and is beyond the scope of this article.

The IPSS [5] includes three prognostic factors—cytogenetic abnormalities, bone marrow blast percentage, and the number of cytopenias—to stratify patients into four distinct risk categories: Low, Intermediate-1, Intermediate-2, and High Risk. The revised version, IPSS-R [6], refines these parameters by expanding the cytogenetic classification from three to five categories, improving blast stratification, and individually evaluating each cytopenia with respect to severity. These refinements yield a five-tiered system: Very Low, Low, Intermediate, High, and Very High Risk. The WPSS [7], on the other hand, incorporates transfusion dependence—an established independent prognostic factor particularly in lower-risk patients [15], which is associated with decreased survival and increased risk of AML transformation [16,17]—alongside WHO classification of MDS, which partially overlaps with blast count and the same cytogenetic stratification as IPSS-R, resulting in the same five categories: Very Low, Low, Intermediate, High, and Very High Risk.

All three prognostic scoring systems facilitate classification into two broader risk categories relevant for therapeutic decision-making: Lower-Risk MDS (LR-MDS)—includes Low and Intermediate-1 risk groups according to IPSS, and Very Low, Low, and Intermediate groups according to IPSS-R and WPSS—and Higher-Risk MDS (HR-MDS)—includes Intermediate-2 and High-risk groups in IPSS, and High and Very High-risk groups in IPSS-R and WPSS.

Risk stratification plays a pivotal role in guiding therapy and anticipating disease progression. Current guidelines [18,19,20] recommend tailoring therapeutic strategies according to patient risk categories. For lower-risk MDS, supportive care is the cornerstone of management. The goals are to reduce transfusion dependence, delay progression to higher-risk disease or AML, and improve survival [21]. First-line treatment for anemia typically includes red blood cell transfusions and erythropoiesis-stimulating agents (ESAs) [18,19]. In ESA-refractory cases, agents such as Luspatercept [22] or Imetelstat [23] may be used. Lenalidomide is indicated in patients with isolated del(5q) [24]. Additional supportive therapies include platelet transfusions, granulocyte colony-stimulating factors, antimicrobial prophylaxis, and treatment of iron overload [25]. For higher-risk MDS, Azacitidine remains the standard of care [26], offering superior overall survival compared to conventional treatment approaches (24 vs. 15 months) [27]. Other therapeutic options include Decitabine [28] and Decitabine/Cedazuridine [29]. Emerging therapies show promise, such as Ivosidenib for IDH1-mutated MDS [30] and Venetoclax in combination with Azacitidine for relapsed or refractory disease [31]. Furthermore, allogeneic stem cell transplantation remains the only potentially curative option and should be considered in younger, fit patients [32,33].

In conclusion, accurate risk stratification is essential in MDS to predict prognosis and guide therapeutic decisions. While IPSS remains the standard for treatment eligibility in some regions, its limitations—particularly in borderline-risk cases—may lead to under-treatment. Wider implementation of IPSS-R or IPSS-M may help address this gap by enabling more precise identification of patients in need of timely and effective intervention.

## 2. Materials and Methods

This single-center retrospective study included patients diagnosed with myelodysplastic neoplasms who were treated in our university medical center over a five-year period (2018–2022). The main inclusion criteria were age over 18 years old and a confirmed diagnosis of MDS based on bone marrow biopsy and cytogenetic examination. Patients who had only dysplasia identified on a bone marrow aspirate without biopsy confirmation, as well as those who did not undergo cytogenetic testing at diagnosis, were excluded from the study, as prognostic scoring could not be applied to these cases. Data were collected from clinical medical records and third-party laboratories responsible for bone marrow biopsy processing and cytogenetic analyses.

Collected variables included epidemiological data (e.g., age, sex), complete blood count parameters, transfusion dependence (defined as the need for four or more units of blood over an eight-week period at diagnosis or at any point during the follow-up period), histopathological and immunohistochemistry findings from the bone marrow including types of cell dysplasia, percentage of bone marrow blasts, percentage of ring sideroblasts and cytogenetic findings. The data were used to calculate three prognostic scores: IPSS, IPSS-R, and WPSS. Patients were then classified into two main prognostic categories based on these scoring systems: LR- (IPSS low and intermediate-1 risk; IPSS-R very low, low, and intermediate risk; and WPSS very low, low, and intermediate risk) and HR-MDS (IPSS intermediate-2 and high risk; IPSS-R high and very high risk; and WPSS high and very high risk) according to the three prognostic scoring systems.

The primary objective of the study was to assess, in a single-center real-world Romanian cohort, how accurately the IPSS, IPSS-R, and WPSS prognostic scores stratify patients with MDS. This was performed by comparing their assigned risk categories with expected overall survival (OS) and time to leukemic transformation (progression-free survival, PFS). Each group of patients (lower-risk versus higher-risk) as defined by each prognostic scoring system was compared to one another in terms of OS, median survival, and PFS using Kaplan–Meier survival analysis with the log-rank test. A *p*-value < 0.05 was considered to be statistically significant. Median OS and PFS were estimated from Kaplan–Meier curves at the time point where the survival probability fell to 0.50. Hazard ratios were estimated using Cox proportional hazards models and are presented with corresponding 95% confidence intervals and *p*-values.

Kaplan–Meier survival curves were computed in Python (lifelines v0.27.4 package) and rendered with Matplotlib (v 3.6.3). Draft vector graphics were further refined with OpenAI’s generative-image model (OpenAI o4-mini-high; OpenAI, San Francisco, CA; USA, 2025) to enhance visual clarity. All graphical outputs were reviewed and manually edited by the authors prior to publication.

## 3. Results

The analysis included a cohort of 117 patients diagnosed and treated in our clinic. Median age at diagnosis was 70 years (range: 31–89 years), with 58 male and 59 female patients. Among these, 11.1% (n = 11) had secondary myelodysplastic syndromes following chemotherapy and/or radiotherapy for other malignancies. Notably, 73.5% (n = 86) of patients were transfusion dependent.

Analysis of cytopenias was conducted based on the criteria defined by the IPSS and IPSS-R scoring systems. According to IPSS, 57 patients (48.7%) had neutropenia, 99 patients (84.6%) had anemia, and 54 patients (46.1%) had thrombocytopenia. When applying IPSS-R criteria, 25 patients (21.4%) were found to have neutropenia, 72 patients (61.5%) had severe anemia (<80 g/L), and 21 patients (18%) had severe thrombocytopenia (<50 × 10^9^/L).

The majority of patients (n = 74; 63.3%) exhibited an excess of bone marrow blasts (>5%), which corresponded to histopathological subtypes defined by the 2016 WHO Classification of myelodysplastic syndromes. Most patients were classified as having refractory anemia with excess blasts type II (n = 38; 32.5%), followed by refractory anemia with excess blasts type I (n = 27; 23.1%) and refractory cytopenia with multilineage dysplasia (n = 24; 20.5%). Medullary fibrosis was present in seven patients (6%), with severity ranging from grade 1 to grade 3.

**Figure 1:**
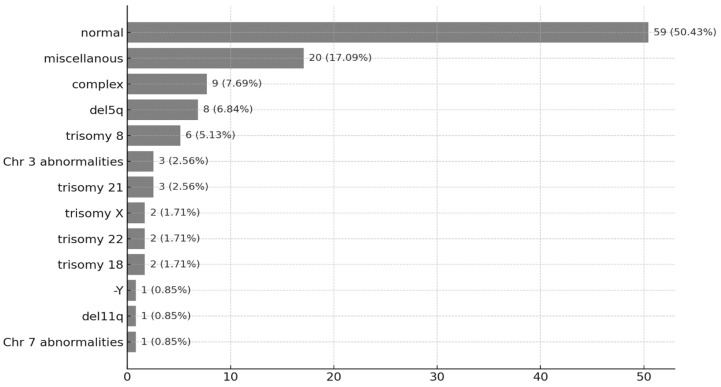
Distribution of karyotype abnormalities.

Cytogenetic analysis revealed that 59 patients (50.4%) had a normal karyotype. Other notable findings included complex karyotype (n = 9; 7.7%), isolated del(5q) (n = 8; 6.8%), and trisomy 8 (n = 6; 5.1%). Cytogenetic risk stratification showed that 69 patients (59%) had favorable-risk cytogenetics, while 32 patients (27.3%) were classified as having intermediate-risk cytogenetics. A detailed summary of the epidemiological, morphological, and cytogenetical findings is presented in Table 1 and Table 2 and Figure 1.

**Figure 2:**
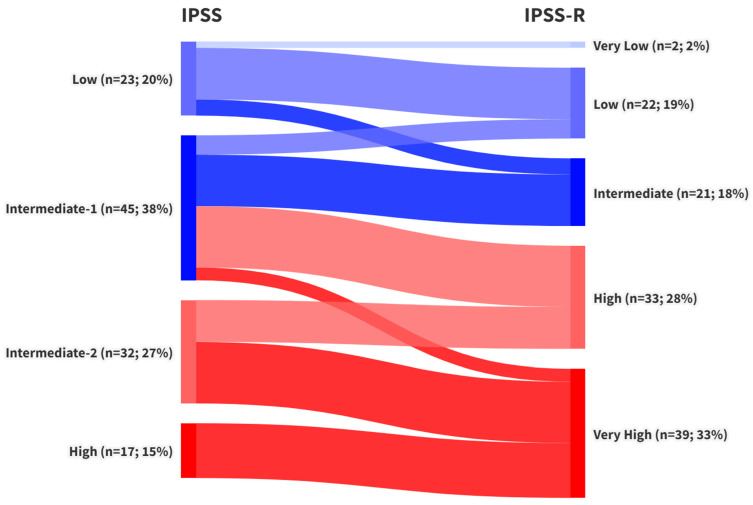
Transition of MDS risk stratification in the study cohort (n = 117) from IPSS to IPSS-R categories.

**Figure 3:**
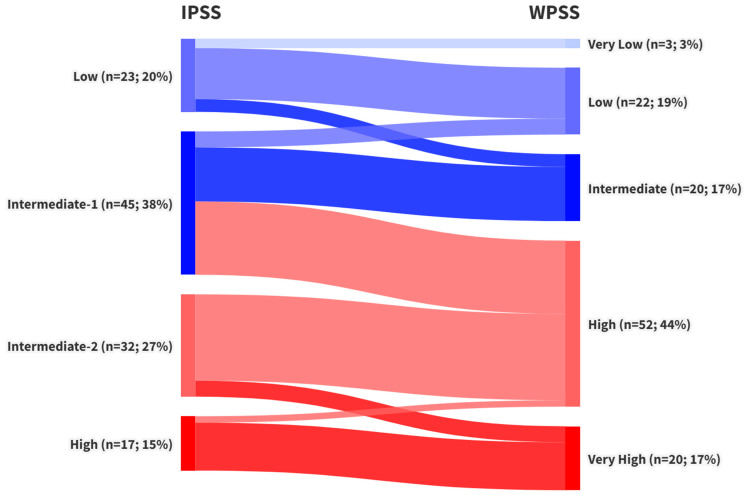
Transition of MDS risk stratification in the study cohort (n = 117) from IPSS to WPSS categories.

Stratification of patients according to the three prognostic scores revealed that 68 patients (58%) were classified as lower-risk and 49 patients (42%) as higher-risk by the IPSS. Under the IPSS-R system, 45 patients (38.5%) fell into the lower-risk group, while 72 patients (61.5%) were classified as higher risk. The WPSS produced identical proportions, with 45 patients (38.5%) in the lower-risk and 72 patients (61.5%) in the higher-risk category. A detailed comparison of these stratifications and the reclassification of patients across scoring systems is presented in Figure 2 and Figure 3.

The OS rate in the cohort was 35.9% (42 of 117 patients), with a median OS of 20 months. Among patients classified as LR-MDS, OS was 52.9% (36/68) with a median survival of 60 months according to the IPSS; 64.4% (29/45) with a median of 109 months based on the IPSS-R; and 62.2% (28/45) with a median of 109 months using the WPSS. No statistically significant differences in survival were observed among the low-risk groups.

In the higher-risk groups, OS was 12.2% (6/49) with a median survival of 12 months according to the IPSS; 18.1% (13/72) with a median of 12 months based on the IPSS-R; and 19.4% (14/72) with a median of 12 months using the WPSS. Similarly, no statistically significant survival differences were observed among these high-risk classifications.

Regarding causes of death, the majority of patients died from infectious complications, most commonly bronchopneumonia (38%) and septic shock (6.5%). Hemorrhagic events were also frequent, with cerebral hemorrhage accounting for 41% and gastrointestinal hemorrhage for 8% of deaths. Cardiac complications were responsible for approximately 5% of fatalities. Notably, around 35% of these complications were associated with bone marrow failure and aggressive leukemic proliferation following leukemic transformation.

Leukemic transformation occurred in 50 patients (42.8%) across the cohort, with a median time to progression of 35 months. Among patients classified as LR-MDS, transformation rates were 25% (17/68) in the IPSS group, 11.1% (5/45) according to the IPSS-R, and 13.3% (6/45) based on the WPSS. The median time to progression was not reached in any of the LR-MDS groups. A statistically significant difference in the risk of leukemic transformation was observed between the IPSS and IPSS-R LR-MDS groups (*p* = 0.045).

In HR-MDS patients, leukemic transformation occurred in 67.4% (33/49) according to the IPSS (median time to progression: 9 months), 62.5% (45/72) based on the IPSS-R (median: 11 months), and 61.1% (44/72) according to the WPSS (median: 12 months). No statistically significant differences were observed among the high-risk groups. A comparison of OS and PFS rates and medians—overall and across risk groups/prognostic scores—is provided in Table 3.

Given the absence of statistically significant differences in OS and PFS across the scoring systems when analyzed separately, a multivariable analysis using the Cox proportional hazards model was conducted to adjust for potential confounding variables. The following covariates were included: age, gender, risk stratification by IPSS, IPSS-R, and WPSS, transfusion dependence, cytogenetic risk group (as defined by IPSS-R), Azacitidine treatment, and baseline levels of lactate dehydrogenase (LDH), ferritin, and albumin.

For OS, stratification by IPSS-R (lower vs. higher risk) was identified as a strong independent predictor of mortality (HR = 3.22; 95% CI: 1.98–5.25; *p* = 0.000003). Treatment with Azacitidine was also associated with a significantly reduced risk of death (HR = 0.43; 95% CI: 0.29–0.64; *p* = 0.00002). Cytogenetic risk, as defined by IPSS-R, demonstrated a trend toward increased mortality (HR = 1.22), though statistical significance was not reached (*p* = 0.072). No statistically significant associations were found for age, IPSS, WPSS, transfusion dependence, LDH, ferritin, or albumin levels.

For PFS, IPSS-R stratification remained a strong independent predictor of leukemic transformation (HR = 4.77; 95% CI: 2.45–9.29; *p* < 0.00001), while Azacitidine treatment was significantly associated with a lower risk of progression (HR = 0.36; 95% CI: 0.16–0.81; *p* = 0.013). Cytogenetic risk again showed a non-significant trend toward increased risk (HR = 1.42; 95% CI: 0.98–2.06; *p* = 0.064). All other variables included in the model did not reach statistical significance.

To further investigate the impact of Azacitidine treatment (n = 47; 40.2%) within clinically relevant subgroups, Kaplan–Meier survival analyses were performed for patients classified as HR-MDS according to each prognostic scoring system.

In the IPSS high-risk subgroup, Azacitidine treatment was associated with a significant improvement in both OS and PFS, with median OS of 14 months in treated patients versus 7 months in untreated patients (*p* = 0.0013), and median PFS of 18 months versus 4 months, respectively (*p* = 0.000003).

For patients in the IPSS-R higher-risk categories, Azacitidine also conferred a significant survival benefit (median OS: 14 vs. 11 months; *p* = 0.0239). A trend toward improved PFS was observed (median 18 vs. 11 months); however, this did not reach statistical significance (*p* = 0.0581).

In the WPSS higher-risk group, Azacitidine treatment significantly prolonged OS (median 15 vs. 12 months; *p* = 0.0257), while no significant difference in PFS was observed between treated and untreated patients (median 16 vs. 24 months; *p* = 0.2266).

During the primary analysis, notable variability was observed in the restratification of patients initially classified as IPSS intermediate-1 (int-1) when reassessed using IPSS-R and WPSS criteria. This group displayed inconsistent redistribution across LR- and HR-MDS categories (Figure 2 and Figure 3). Median OS for IPSS int-1 patients was 31 months, compared to 111 months for the IPSS low-risk group and 90 months for the combined LR-MDS category (IPSS low- and intermediate-1-risk patients). Due to this heterogeneity, these 45 patients were analyzed separately. Among them, 22 were reclassified as LR-MDS and 23 as HR-MDS under both IPSS-R and WPSS.

Outcome analysis revealed a median OS of 67.5 months and a survival rate of 45.5% (10/22) in the IPSS int-1/IPSS-R LR-MDS group, compared to 15 months and 34.8% (8/23) in the IPSS int-1/IPSS-R HR-MDS group (HR = 0.24; 95% CI: 0.10–0.59; *p* = 0.0017). Nearly identical results were obtained with WPSS stratification, with the IPSS int-1/WPSS HR-MDS group showing a median OS of 13 months (HR = 0.26; 95% CI: 0.10–0.63; *p* = 0.0031). These differences were statistically significant for both IPSS-R (*p* = 0.0007) and WPSS (*p* = 0.0015).

Regarding leukemic transformation, the IPSS int-1/IPSS-R LR-MDS group had a transformation rate of 13.6% (3/22), compared to 52.2% (12/23) in the HR-MDS group. WPSS yielded similar results, with transformation rates of 18.2% (4/22) and 48.2% (11/23) in the LR- and HR-MDS groups, respectively. Median PFS was not reached in either LR-MDS group, while it was 21 months in both HR-MDS groups. These differences were statistically significant based on log-rank tests for both IPSS-R (*p* = 0.0003) and WPSS (*p* = 0.0003). Cox proportional hazard models further confirmed these findings, with HR = 0.13 (95% CI: 0.04–0.48; *p* = 0.0021) for IPSS-R and HR = 0.12 (95% CI: 0.03–0.47; *p* = 0.002) for WPSS.

## 4. Discussion

Prognosis in myelodysplastic neoplasms is highly heterogeneous, ranging from lower-risk patients who may require only supportive care to higher-risk patients who benefit from more intensive therapies, such as hypomethylating agents, AML-like induction chemotherapy, or allogeneic stem cell transplantation. Therefore, accurate risk stratification is essential for guiding individualized treatment decisions in MDS. In this study, we aimed to compare prognostic risk stratification tools in a cohort of patients diagnosed and treated at our center. To our knowledge, this is the first study of its kind conducted on a Romanian patient population.

The cohort consisted of 117 patients, with a median age at diagnosis of 70 years—consistent with large epidemiological studies and the well-documented increase in MDS incidence with advancing age [34,35]. This age-related trend was also reflected in our population, with the highest proportion of cases (41%) occurring in the 70–79 age group. Gender distribution was nearly equal, in line with existing literature that reports either a balanced sex distribution or a slight male predominance [6].

In terms of cytopenia assessment, anemia was the most prevalent cytopenia (84.6%), followed by neutropenia (48.7%) and thrombocytopenia (46.1%) based on IPSS criteria. These findings are consistent with previous studies indicating that anemia is the most common cytopenia in MDS [36]. When re-evaluated using IPSS-R thresholds, severe anemia was present in 61.5% of patients, severe thrombocytopenia (<50 × 10^9^/L) in 18%, and neutropenia in 21.4%. These results underscore the importance of assessing not only the presence but also the severity of cytopenias, as emphasized in the IPSS-R [6]. This approach enables improved prognostic stratification and supports individualized treatment decision-making. In our analysis, transfusion dependence was present in 73.5% of patients, which is in accordance with literature data that suggests that up to 90% of patients with MDS will receive transfusions during their clinical course, and as many as 70% of patients will become transfusion-dependent [37].

An important finding in our cohort was the strong correlation between cytopenias and both clinical complications and reduced quality of life. Severe anemia (hemoglobin levels < 80 g/L) was present in 72 patients (61.5%) at diagnosis, and its progression contributed to the high rate of transfusion dependence (73.5%). A small subset of patients (n = 12; 9%) with RARS-MDS received treatment with ESAs, but responses were limited, with only five patients achieving transfusion independence. Frequent transfusions led to iron overload in nearly half of the patients (46%), necessitating long-term iron chelation therapy. Both transfusion dependence and secondary iron overload significantly impaired patient quality of life by increasing hospitalization frequency and reducing functional capacity. Cardiac complications attributable to iron overload were a direct cause of death in three patients.

Additionally, moderate to severe neutropenia (defined as an absolute neutrophil count < 1.0 × 10^9^/L) was observed in approximately one-third of patients (n = 37; 31.5%) and was strongly associated with a high incidence (44.5%) of infectious complications. As neutropenia worsened, infection rates rose further, contributing to prolonged hospital stays and diminished quality of life. As previously noted, infectious complications were the leading cause of mortality in our study cohort.

Cytogenetic abnormalities were identified in 49.6% of patients, aligning with data reported in previous studies [38] and falling within the expected range for MDS populations. The most frequently observed abnormalities were complex karyotype (7.7%), isolated deletion 5q (6.8%), and trisomy 8 (5.1%) [39]. Cytogenetic findings remain among the most important prognostic indicators in MDS. To enhance prognostic precision, the IPSS-R introduced five distinct cytogenetic risk categories, allowing for more refined risk stratification [6].

In our study, the incorporation of these five IPSS-R cytogenetic risk categories into the multivariable analysis underscored their prognostic significance. Although these categories did not reach conventional statistical significance, they demonstrated a consistent trend toward increased hazard for both OS and PFS.

More recently, the development of the IPSS-M has further enhanced risk stratification by integrating somatic mutational data, thereby enhancing our understanding of disease biology and prognosis [8]. However, the routine implementation of IPSS-M in clinical practice remains limited due to its high cost and the restricted availability of the molecular testing techniques.

After stratifying patients into LR- and HR-MDS categories according to IPSS, IPSS-R, and WPSS—and analyzing risk category shifts across these prognostic systems (see Figure 2 and Figure 3)—we examined the study’s two primary endpoints: overall and progression-free survival. The median OS in the overall cohort was 20 months, consistent with published data reporting median survival in unstratified MDS populations ranging from 18 to 30 months [35,40,41]. The three-year survival rate was 41.1%, closely aligning with findings from larger cohorts reporting a 3-year OS of 42.2% [41]. Other studies have noted median OS values of 14 to 15 months in untreated patients and 20 to 24 months in those treated with Azacitidine [27,42]. Median OS values for LR- and HR-MDS groups were consistent with those reported in the original validation studies for the IPSS [5], IPSS-R [6], and WPSS [7].

Regarding progression-free survival (i.e., time to leukemic transformation), the median time in our overall cohort was 35 months. Direct comparisons with existing literature are challenging, as most studies report data stratified by LR- and HR-MDS groups rather than across unstratified populations. In previously published cohorts, the median time to AML progression is reported as approximately 29 months in LR-MDS and 22 months in HR-MDS [43]. In our own LR-MDS subgroup, the median PFS was not reached. By contrast, the HR-MDS subgroup showed median PFS ranging from 9 to 12 months depending on the scoring system used. These findings are consistent with previously published data reporting a median PFS of 11 to 13 months without hypomethylating therapy and 18 to 21 months with Azacitidine treatment [27,42].

Differences in OS and PFS between patients who received Azacitidine and those who did not were also observed in our cohort. Azacitidine treatment emerged as one of only three factors independently associated with both survival and progression in the multivariable Cox regression analysis. The most pronounced and statistically significant differences in OS and PFS were observed within the IPSS-defined HR-MDS group, while the effects were less robust for IPSS-R and WPSS, particularly in relation to PFS.

We believe that this attenuation of statistical significance may be explained by national treatment guidelines, which mandate that Azacitidine be offered exclusively to patients classified as HR-MDS based on IPSS criteria. Consequently, some patients categorized as higher-risk by IPSS-R or WPSS—but stratified as lower-risk by IPSS—were not eligible to receive Azacitidine. This discrepancy likely led to poorer outcomes in these patients and introduced a bias that diminished the observed treatment effect in the IPSS-R and WPSS analyses.

In terms of direct comparison, existing literature generally supports the superiority of IPSS-R. One study reported that IPSS-R outperformed IPSS in predicting OS [44], with a C-index of 0.74 (*p* < 0.001), and showed borderline significant superiority over WPSS (*p* = 0.05), although no significant differences were found for PFS prediction. The original IPSS-R study [6] demonstrated better risk discrimination compared to IPSS, with higher Dxy values for both survival (0.43 vs. 0.37) and AML evolution (0.52 vs. 0.48). Additionally, another analysis confirmed that WPSS was significantly superior to IPSS in predicting PFS (*p* < 0.001), while both WPSS (*p* < 0.001) and IPSS-R (*p* = 0.037) showed better performance than IPSS in predicting OS [45].

However, our findings did not replicate these results. No statistically significant differences were identified between IPSS, IPSS-R, and WPSS in terms of OS or PFS, except for a single significant difference in PFS between IPSS and IPSS-R among LR-MDS groups (*p* = 0.045). Despite this, IPSS-R emerged as the strongest independent predictor of both OS and PFS in the multivariable Cox regression analysis, suggesting a potentially greater prognostic utility compared to IPSS and WPSS.

Notable discrepancies also emerged when comparing survival outcomes across the scoring systems. Specifically, median OS for IPSS-defined LR-MDS patients was 60 months, whereas IPSS-R and WPSS-defined LR-MDS patients had a median OS of 109 months. Although these differences were not statistically significant, they prompted further investigation. A detailed review of reclassification patterns (Figure 2 and Figure 3) revealed that patients initially classified as IPSS int-1 were variably reassigned as either LR- or HR-MDS by IPSS-R and WPSS. This finding contrasts with the original IPSS-R study [6], which suggested that most IPSS int-1 patients are reclassified as Very Low or Low IPSS-R risk (LR-MDS).

**Figure 4:**
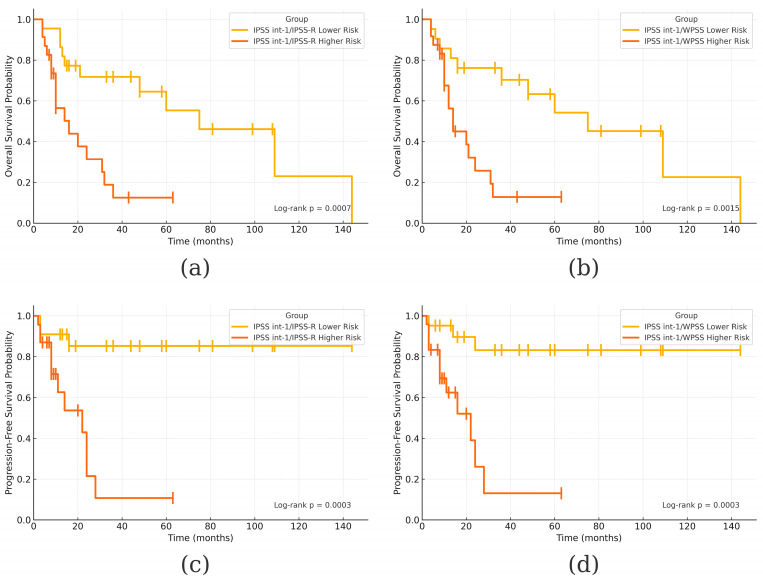
Kaplan–Meier OS and PFS in IPSS int-1 patients after re-classification into Lower Risk and Higher Risk groups by IPSS-R and WPSS: (**a**) OS after re-classification by IPSS-R; (**b**) OS after re-classification by WPSS; (**c**) PFS after re-classification by IPSS-R; (**d**) PFS after re-classification by WPSS. Vertical bars (“|”) indicate censored observations. Log-rank tests were used for pairwise survival comparisons. Reported *p*-values are unadjusted for multiple comparisons. However, all statistically significant results remained significant after applying Bonferroni, Holm, and Benjamini–Hochberg corrections.

Additionally, the observed difference in PFS between IPSS and IPSS-R LR-MDS patients (*p* = 0.045) likely reflects the prognostic ambiguity inherent to the IPSS int-1 risk category. Consequently, we performed a separate analysis for this subgroup. When IPSS int-1 patients were reclassified using IPSS-R and WPSS, significant differences in both OS and PFS emerged between those reallocated to lower- versus higher-risk categories, as shown by Kaplan–Meier analyses (Figure 4).

These findings are partly supported by literature data. Warlick et al. [46] noted that patients classified as IPSS int-1 and int-2 were inconsistently redistributed across the IPSS-R spectrum, ranging from Very Low to High Risk, although the implications of this redistribution for OS and PFS were not directly assessed. Benton et al. [9] specifically examined the IPSS-R intermediate-risk group and showed that clinical factors such as age over 66 years, peripheral blood blasts ≥ 2%, and prior red blood cell transfusion significantly enhanced prognostic discrimination. Another study [47] also emphasized that comorbidities exert an independent prognostic influence on survival, beyond established scoring systems.

Taken together, these observations reinforce the notion that existing prognostic tools, while essential, are not infallible. Stratification tools like IPSS-R and WPSS may benefit from additional refinement or supplementation with clinical and molecular predictors. In particular, applying more nuanced stratification to IPSS int-1 patients can reveal significant differences in outcomes and help guide access to appropriate treatment, especially in healthcare systems where treatment eligibility is still tied to IPSS risk categories.

The absence of statistically significant survival differences across IPSS, IPSS-R, and WPSS risk groups in our cohort contrasts with previously published findings. This discrepancy may be partly explained by treatment allocation practices shaped by national policy, which authorizes Azacitidine use only for patients categorized as higher-risk according to IPSS. Consequently, patients considered higher-risk by IPSS-R or WPSS but classified as low-risk by IPSS did not receive Azacitidine and experienced inferior outcomes. This introduced a confounding bias that likely contributed to the attenuation of survival differences. Additionally, risk group discordance—particularly the reclassification of a large proportion of IPSS intermediate-1 patients into higher- or lower-risk groups by IPSS-R and WPSS—may have diluted the prognostic contrast between strata. Finally, limited sample sizes within individual risk subgroups may have reduced the statistical power to detect significant differences in overall and progression-free survival. These factors, taken together, may explain the lack of concordance between our results and those reported in the literature.

This study has several limitations inherent to its retrospective, single-center design. First, treatment decisions—particularly the administration of Azacitidine—were influenced by national policy, which restricted its use to patients classified as high-risk according to IPSS. This introduced treatment allocation bias and likely affected survival outcomes across other scoring systems, such as IPSS-R and WPSS. Second, the relatively small sample size within individual risk groups may have limited the statistical power to detect survival differences, particularly in subgroup analyses. Third, the real-world nature of the cohort introduces potential heterogeneity in patient management, follow-up, and data completeness, especially for laboratory parameters with missing values which may have influenced multivariable model robustness despite the use of multiple imputation. Lastly, the study did not incorporate molecular genetic data, which are increasingly important for risk stratification in myelodysplastic neoplasms and may have added further prognostic resolution.

To our knowledge, no previous studies have directly examined the impact on OS and PFS of patients initially classified as IPSS int-1 when reclassified using either IPSS-R or WPSS alone. Although we could not identify external data to validate our results, we consider these findings particularly significant within the context of our national healthcare system. Under current local guidelines and reimbursement policies, access to hypomethylating agents is restricted to patients stratified as intermediate-2 or high-risk according to the IPSS. This presents a critical challenge, as the IPSS may inadequately stratify borderline cases—particularly those falling between intermediate-1 and intermediate-2 risk—potentially causing higher-risk patients to be misclassified within the intermediate-1 category and rendering them ineligible for treatment despite clinical need. This highlights a critical limitation of the IPSS in accurately capturing risk among borderline cases.

In light of these findings, we advocate for a revision of national reimbursement criteria to incorporate IPPS-R as the preferred risk stratification tool. While full integration of molecular data into clinical practice may not yet be feasible in all settings, the interim adoption of IPSS-R—which offers better stratification and improved prognostic accuracy—represents an immediately actionable strategy. Such a revision would align treatment eligibility more closely with current international guidelines and ensure that high- and very high-risk patients are not excluded from receiving hypomethylating agents such as Azacitidine.

## 5. Conclusions

This study highlights the variability in patient stratification between IPSS, IPSS-R, and WPSS and its clinical implications in myelodysplastic neoplasms. Although no statistically significant differences in OS or PFS were observed when directly comparing these scoring systems, IPSS-R emerged as the strongest independent predictor in multivariable analysis, suggesting enhanced prognostic utility. A particularly relevant finding was the reclassification of patients initially identified as IPSS intermediate-1, many of whom were reassigned to higher-risk categories under IPSS-R or WPSS. These patients exhibited poorer outcomes, which were not reflected in IPSS-based assessments.

Importantly, national treatment policy that restricts Azacitidine access exclusively to patients deemed high-risk by IPSS may have led to suboptimal treatment in patients more accurately stratified as high-risk by IPSS-R or WPSS. This policy-related treatment gap likely contributed to the diminished survival statistics observed in these scoring systems. Our findings strongly support the need to revise national guidelines and reimbursement frameworks to incorporate IPSS-R as the preferred stratification tool, thereby improving therapeutic allocation and aligning patient management with contemporary prognostic standards.

## Figures and Tables

**Table 1:** Baseline characteristics of the study cohort.

	n	%	Median
**Age**			70 [31–89]
30–39	4	3.5	
40–49	4	3.5	
50–59	6	5	
60–69	40	34	
70–79	48	41	
≥80	15	13	
**Sex**			
Male	58	49.5	
Female	59	50.5	
**Neutrophil count (×10^9^/L)**			1.89 [0.11–5.31]
>1.8	60	30	
0.8–1.8	32	49.5	
<0.8	25	21.5	
**Hemoglobin (g/L)**			7.3 [4–14.5]
≥100	18	15.5	
80–100	27	23	
<80	72	61.5	
**Platelets (×10^9^/L)**			113 [5–697]
>100	63	54	
50–100	33	28	
<50	21	18	
**Transfusion dependence** **^1^**	86	73.5	
**Medullary Blasts (%)**			
0–2	26	22	
>2–<5	17	14.5	
5–10	36	31	
>10	38	32.5	
**WHO Morphological Category** **^2^**			
RA	4	3	
RARS	15	13	
RCMD	24	20.5	
del5q	8	7	
RAEB-1	27	23	
RAEB-2	38	32.5	
Hypoplastic	1	1	

^1^ Transfusion dependence defined as 4 or more units of blood required over a period of 8 weeks; ^2^ Abbreviations: RA, Refractory Anemia; RARS, Refractory Anemia with Ringed Sideroblasts; RCMD, Refractory Cytopenia with Multilineage Dysplasia; del5q, Myelodysplastic Syndrome with Isolated Del(5q); RAEB-1, Refractory Anemia with Excess of Blasts type 1; RAEB-2, Refractory Anemia with Excess of Blasts type 2.

**Table 2:** Karyotype risk categories as defined by IPSS-R.

Category	n	%
Very Good	2	1.7
Good	69	59
Intermediate	32	27.3
Poor	5	4.3
Very Poor	9	7.7

**Table 3:** Overall and progression-free survival rates and median times by IPSS, IPSS-R, and WPSS risk categories.

Category	OS Rate	OS Median	PFS Rate	PFS Median
Global	42/117 (35.9%)	20 months	50/117 (42.7%)	35 months
IPSS Lower Risk	36/68 (53%)	60 months	17/67 (25%)	Not reached
IPSS-R Lower Risk	29/45 (64.4%)	109 months	5/45 (11.1%)	Not reached
WPSS Lower Risk	28/45 (62.2%)	109 months	6/45 (13.3%)	Not reached
IPSS Higher Risk	6/49 (12.3%)	12 months	33/49 (67.4%)	9 months
IPSS-R Higher Risk	13/72 (18%)	12 months	45/72 (62.5%)	11 months
WPSS Higher Risk	14/72 (19.4%)	12 months	44/72 (61.1%)	12 months

OS, overall survival—defined as time from diagnosis to event (death); PFS, progression-free survival—defined as time to event (leukemic transformation). Median overall survival (OS) and progression-free survival (PFS) were estimated from Kaplan–Meier curves, with the medians taken at the time point where the survival probability fell to 0.50. All median survival values were calculated using the same endpoint and applying a consistent censoring strategy across analyses.

## Data Availability

The original contributions presented in this study are included in the article. Further inquiries can be directed to the corresponding author.

## Abbreviations

The following abbreviations are used in this manuscript:

AML	Acute Myeloid Leukemia
CI	Confidence Interval
Del(5q)	5q Chromosome Deletion
ESAs	Erythropoietin Stimulating Agents
HR	Hazard Ratio
HR-MDS	Higher-Risk Myelodysplastic Syndromes
HSC	Hematopoietic Stem Cells
IDH1	Isocitrate Dehydrogenase 1
IPSS	International Prognostic Scoring System
IPSS int-1	Intermediary-1 IPSS Group
IPSS-M	Molecular International Prognostic Scoring System
IPSS-R	International Prognostic Scoring System-Revised
LDH	Lactate Dehydrogenase
LR-MDS	Lower-Risk Myelodysplastic Syndromes
MDS	Myelodysplastic Syndromes
OS	Overall Survival
PFS	Progression-Free Survival
WHO	World Health Organization
WPSS	WHO Prognostic Scoring System
